# Total Hip Arthroplasty: So Hip It Hurts

**DOI:** 10.3390/jcm12113849

**Published:** 2023-06-05

**Authors:** Lukas A. Holzer

**Affiliations:** 1 Department of Orthopaedics, Fiona Stanley Fremantle Hospitals Group, Murdoch, WA 6150, Australia; info@ortho-holzer.com; 2 Perth Orthopaedic and Sports Medicine Centre, West Perth, WA 6005, Australia

Total hip arthroplasty (THA) has become a standard surgical intervention for patients with hip joint disorders [1]. The pioneering work of Sir John Charnley, considered the “father of hip arthroplasty”, laid the foundation for modern THA and has resulted in significant improvements in clinical outcomes. His low-friction arthroplasty technique using metal and polyethylene components, along with the use of cement for fixation, revolutionized hip replacement surgery and significantly improved implant survival rates [2]. Since the times of Charnley’s practice, significant advancements have been made in surgical planning and techniques; implant design, materials, and perioperative care resulted in improved patient outcomes [1,3]. Numerous studies have demonstrated the efficacy and safety of THA in relieving pain, improving function, and enhancing quality of life for patients with hip joint disorders [4,5].

In recent years, there have been significant advancements in surgical techniques for hip arthroplasty. Minimally invasive approaches, such as the direct anterior approach, have gained popularity due to their potential benefits, which include reduced blood loss, shorter hospital stays, and faster recovery [6]. New technologies, such as computer-assisted and robotic-assisted surgery, have also shown promising results in improving accuracy and precision in implant positioning, potentially leading to better outcomes in terms of implant survival and patient satisfaction [7,8].

Implant design has also evolved in recent years to improve the survival and performance of THA. Advances in materials science have led to the development of highly durable and biocompatible implant materials, such as ceramics and highly crosslinked polyethylene, which have shown promising results in reducing wear and implant failure rates [9]. Additionally, implant designs have been optimized to improve biomechanics and stability, with features such as modular dual-mobility bearings and short stems [10,11].

It should be noted that certain developments in this field, such as metal-on-metal (MoM) bearings, were initially believed to be game changers. However, they were found to have high revision rates due to adverse reactions to metal debris and increased serum metal ions. As a result, MoM bearings are hardly used nowadays despite their initial promise [12,13].

Perioperative management has also been optimized in recent decades to improve outcomes in THA. Enhanced recovery after surgery (ERAS) protocols have been implemented in many centers, which involve preoperative optimization, intraoperative measures to reduce surgical stress, and postoperative rehabilitation programs to facilitate early recovery [14]. These protocols have been shown to reduce complications, shorten hospital stays, and improve patient satisfaction.

While THA has come a long way, there are several areas of research that are promising for the future of this field. Some of the key future perspectives include:

1. Personalized medicine: Personalized medicine, including genomics, could potentially play a role in THA by tailoring the surgical approach, implant design, and postoperative care to individual patients. Genetic profiling could help identify patients who may be at increased risk for implant failure or complications, allowing for a more customized management [15]. Additionally, advances in 3D printing and computer modeling could enable the creation of patient-specific implants that better match the patient’s anatomy, potentially leading to improved outcomes and reduced complications [16];
2. Biomaterials and implant coatings: Further advancements in biomaterials and implant coatings could lead to improved implant survival and reduced wear rates. Research is ongoing in developing materials with enhanced biocompatibility, antibacterial properties, and improved lubrication properties to reduce friction and wear. Nanotechnology is also being explored to create surface coatings that promote bone integration and reduce the risk of implant loosening [17]. These advancements could potentially lead to longer-lasting implants with reduced revision rates;
3. Robotics and artificial intelligence: Robotics and artificial intelligence (AI) have the potential to revolutionize THA by improving the accuracy and precision of implant positioning, reducing complications, and optimizing patient outcomes. Robotic-assisted surgery systems have already been developed and are being used in some centers to assist surgeons in performing THA with increased accuracy and reproducibility [7]. AI algorithms are also being developed to analyze large amounts of data, including patient-specific factors, surgical techniques, and implant outcomes, to optimize surgical planning and decision making [18]. These technologies have the potential to improve the long-term success of THA and reduce the need for revisions;
4. Enhanced rehabilitation strategies: Rehabilitation strategies play a crucial role in the success of any surgery. Advances in rehabilitation techniques, such as early mobilization, prehabilitation, and tele-rehabilitation, could further optimize patient outcomes [14]. Tele-rehabilitation, in particular, has gained attention during the COVID-19 pandemic, as it allows for remote monitoring and guidance of patients’ rehabilitation progress, potentially improving access to care and reducing the need for in-person visits [19,20]. Additionally, wearable devices and sensor technologies could be utilized to monitor patient progress, provide real-time feedback, and optimize rehabilitation protocols.

In summary, THA is a well-established treatment option for patients with osteoarthritis of the hip and other hip joint conditions. Advances in implant technology, materials, surgical techniques, and perioperative management strategies have led to improved outcomes and patient satisfaction. Future perspectives in THA include continued improvements in implant materials, designs, and surgical techniques to further enhance the long-term performance and durability of implants. Personalization of implants and the use of new technologies such as 3D printing and artificial intelligence are expected to optimize preoperative planning and implant selection. However, challenges such as young or old age and the increasing prevalence of obesity and comorbidities in patients being considered for THA, as well as optimizing long-term monitoring and patient care need to be addressed. Overall, THA is a constantly evolving field with promising future prospects to further improve the quality of life of patients with hip joint conditions.
